# Transparency and translucency indices for 1,525 pictograms from the Aragonese Portal of Augmentative and Alternative Communication

**DOI:** 10.3389/fpsyg.2024.1467796

**Published:** 2024-10-14

**Authors:** Emiliano Díez, Antonio M. Díez-Álamo, María A. Alonso, Dominika Z. Wojcik, Angel Fernandez

**Affiliations:** ^1^Faculty of Psychology, Institute on Community Integration (INICO), University of Salamanca, Salamanca, Spain; ^2^Institute on Neuroscience (IUNE), University of La Laguna, Tenerife, Spain

**Keywords:** pictograms, ARASAAC, iconicity, Augmentative and Alternative Communication, cognitive accessibility

## Abstract

This study investigated the transparency and translucency of 1,525 pictograms from the Aragonese Portal of Augmentative and Alternative Communication (ARASAAC). A total of 521 participants took part in tasks that involved providing the word that best described the meaning of a pictogram or rating the relationship between a pictogram and a verbal label. This process allowed us to obtain indices of transparency (the quality of pictograms that makes their meaning easily “guessable” in the absence of their referent) and translucency (the degree of perceived relationship between the pictogram and its referent when the latter is present) which were further analyzed to assess their reliability and comparability with similar studies. Additionally, the relationship of those indices with various visual and psycholinguistic characteristics was explored, particularly focusing on the match between the original ARASAAC pictogram names and the most frequently provided names by the participants (modal names) for the pictograms. Results showed relatively low levels of transparency, as well as high levels of translucency, with nouns displaying the highest values in both metrics. For transparency and translucency, word imageability and concreteness were the most correlated factors, and, together with age of acquisition, they were the most important features related to the name matching with ARASAAC. The norms derived from this study enhance our understanding of pictogram perception, empowering stakeholders to leverage data-driven insights for the development and implementation of pictographic systems, thus improving cognitive accessibility.

## 1 Introduction

A pictogram is a symbol, drawing, or graphic icon that transmits an idea or message based on its resemblance to a certain referent. Due to its simplicity and effectiveness, pictograms are used in a wide variety of contexts, such as medicine, pharmacy, road signage, public information, work environments, information technology, etc. An important characteristic of pictograms is that they allow to transmit a message in the absence of a verbal code, which constitutes an advantage with respect to the use of labels or written text, as pictograms eliminate language barriers, and can be easily interpreted by people with limited linguistic abilities or with certain visual problems, in addition to transmitting the message more quickly and capturing better the attention of people (Tijus et al., 2007). Also, pictograms reduce the cognitive load of the receiver, as their pictorial representation favors the synthesis of information and memorization (Vezin, 1984). Due to these significant advantages, pictographic systems have important applications for signage in public or private spaces, or in augmentative and alternative communication (AAC) systems, allowing for improved communicative function in people with intellectual disabilities or with any type of disorder or difficulty that hinders, temporarily or permanently, their communication and use of verbal language (Beukelman and Mirenda, 2005).

Several sets and catalogues of pictograms are available nowadays to be used for different purposes, such as Blissymbols (Bliss, 1965), Rebus (Woodcock et al., 1969), Picture Communication Symbols (PCS) (Mayer-Johnson, 1981), Pictogram Ideogram Communication Symbols (PIC) (Maharaj, 1980), and Picsyms (Carlson, 1985). We want to make special mention to the Aragonese Portal of Augmentative and Alternative Communication (ARASAAC) (Palao, 2018), a large catalog of pictograms, both in black and white and in color, which are often used for a variety of applied purposes in the fields of education, health, signage, software design, and AAC. The pictograms were created by Sergio Palao for ARASAAC,^1^ are freely distributed under a Creative Commons License (BY-NC-SA), and are property of the Aragón Government (Spain). The ARASAAC pictograms are widely used worldwide, as the search words of the catalog have been translated into a large number of languages.

Importantly, pictograms vary in their iconicity, that is, in the degree of relationship between the symbol and its referent (Schlosser and Sigafoos, 2002). Ensuring a high degree of iconicity is essential to guarantee that a pictogram is adequate, useful, and effective, as iconicity determines the extent to which pictograms are understandable. In addition, according to the iconicity hypothesis (Lloyd and Fuller, 1990), the more a symbol resembles its referent, the easier it is learned. Consequently, it is necessary to evaluate pictograms’ iconicity in order to know their potential usefulness and effectiveness, a need that has been addressed by several studies (e.g., Bloomberg et al., 1990; Mirenda and Locke, 1989; Mizuko, 1987; for a review, see Schlosser and Sigafoos, 2002).

However, despite these remarkable efforts to evaluate and compare the iconicity of diverse pictographic systems, very few studies have examined the iconicity of the ARASAAC catalog to date, although its use is extended worldwide. Thus, Cabello and Bertola (2012, 2015) examined the iconicity of 38 ARASAAC pictograms in an adult population, finding adequate levels of iconicity, which were in general superior when compared with the equivalent pictograms in the PCS (Mayer-Johnson, 1981) and the Blissymbols (Bliss, 1965) systems. Bertola (2017) also showed that the iconicity of the ARASAAC pictograms is in general superior to that of PCS and Blissymbols when evaluated in three different population groups, namely adults, children with typical development, and children with autism spectrum disorders (ASD). In addition, Cabello Luque and Mazón Morillas (2018) studied a sample of young children diagnosed with language delay, specific language impairment, or ASD, and showed that a set of 30 pictograms from the ARASAAC catalog not only had higher levels of iconicity than the equivalent pictograms in PCS and Blissymbols, but they were also easier to learn, a finding that is in line with the iconicity hypothesis. Finally, Paolieri and Marful (2018) collected norms of name agreement, H index, number of tip-of-the-tongue states, image agreement, conceptual familiarity, naming response times, and visual complexity for the 295 most frequent items from the ARASAAC catalog, thus providing highly valuable information on the properties and usefulness of the ARASAAC pictograms.

With the aim of both significantly increasing the number of evaluated pictograms and adding finer dimensions to the characterization of these stimuli, in the present study, we evaluated the iconicity of a set of 1,525 pictograms from the ARASAAC catalog, which constitutes the largest set of ARASAAC pictograms analyzed to date. Operationally, pictograms’ iconicity is usually assessed in terms of transparency and translucency. *Transparency* (sometimes called “guessability”) is conceptualized as a quality of pictograms that makes their meaning easily “guessable” by the user, in the absence of their referents (Fuller and Lloyd, 1991; Lloyd and Fuller, 1990). Thus, according to the International Organization for Standardization (ISO), a symbol is considered valid only if 67% of the people are able to correctly understand its meaning (International Organization for Standardization, 2014). Following previous studies (Berthenet et al., 2016; Mok et al., 2015; Roberts et al., 2009), transparency was evaluated by asking participants to write a word, either a verb, a noun, or an adjective, that best described the meaning of each pictogram. On the other hand, *translucency* is defined as a variable that reflects the degree of relationship between the pictogram and its referent, when the latter is present (Fuller and Lloyd, 1991; Lloyd and Fuller, 1990). In line with previous studies (Berthenet et al., 2016; Bloomberg et al., 1990; Lloyd and Fuller, 1990; Mok et al., 2015; Roberts et al., 2009), translucency ratings were obtained by asking participants to assess the degree of relationship between the pictogram and the meaning of a reference word written below each pictogram, using a 7-point scale. In addition, we also provide important descriptive information, including estimations of the reliability and validity of the obtained indices, as well as comparisons with the indices reported in previous studies using pictograms from the ARASAAC database.

The pictograms in the present study were selected by virtue of the availability of objective and subjective psycholinguistic indices for their word referents, a circumstance that will allow for the integration of the newly acquired data with existing normative data regarding other potentially important variables for the comprehension, learning, and retention of the pictograms, such as semantic features, frequency, lexical decision times, age of acquisition, concreteness, familiarity, and perceptual and motor attributes of their word referents. As a result, researchers could use this vast repertoire of systematic information to carry out more specific and detailed selection of materials for studies in diverse fields, such as psycholinguistics, memory, education, signage, software design, or AAC. Furthermore, the comprehensive dataset derived from this study also opens avenues for investigating how visual and linguistic properties influence each other and the overall perception and utility of the pictograms, enabling researchers and practitioners to explore the factors that correlate with transparency or translucency in pictograms and how these factors impact their effectiveness in various contexts.

## 2 Method

### 2.1 Participants

A total of 254 students from the University of Salamanca, Spain, participated in the transparency task. All data from six participants were discarded because their native language was not Spanish. For control purposes, twenty pictograms were presented twice to each participant, and Levenshtein’s distance was calculated between each participant’s responses to each pair of equal pictograms. The mean Levenshtein’s distance across the 20 pairs of equal pictograms was less than 1 for every participant, therefore, no participants were excluded due to inconsistency in their responses. Thus, the transparency sample finally included 248 participants (199 female, 49 male), native Spanish speakers, with a mean age of 19.2 years (*SD* = 2.2; range = 17–30 years).

A total of 267 students from the Universities of Salamanca and La Laguna, both in Spain, participated in the translucency rating task. All data from nine participants whose native language was not Spanish were discarded, and all data from five other participants were eliminated due to inadequate performance in the task, as their mean ratings in the 7-point scale to 20 nonrelated word-pictogram pairs included for control purposes was higher than 3.5. Furthermore, the absolute difference between each participant’s ratings to 20 word-pictogram (related) pairs presented twice for control purposes was calculated. The mean difference across the 20 pairs was less than 1 for every participant, and therefore, no participants were excluded due to inconsistency in their responses. Thus, the translucency sample finally included 253 participants (214 female, 39 male), native Spanish speakers, with a mean age of 19.8 years (*SD* = 1.9; range = 18–34 years). All participants in the study provided informed consent and received course credit for their contribution.

### 2.2 Stimuli

A total of 1,525 pictograms and their corresponding word referents were selected from the ARASAAC catalog^2^ to form the stimuli set studied in both the transparency task and the translucency task. As explained above, the selection criterion for the pictograms was the availability of a substantial set of objective and subjective psycholinguistic indices for their Spanish word referents, such as lexical decision times (González-Nosti et al., 2014) and perceptual and motor attributes (Díez-Álamo et al., 2018).

### 2.3 Procedure

The experimental sessions took place in a quiet large room, where groups of 15 to 25 participants performed their task at a time, using individual computers. The total duration of these sessions was between 45 and 60 min.

The transparency task was implemented by means of an application programmed in jsPsych (de Leeuw, 2015), a JavaScript library for conducting behavioral experiments in a Web browser. The 1,525 stimuli were randomly distributed in three subsets of 381 and one subset of 382 stimuli, and each subset was assigned to a separate transparency subtask. In addition, twenty pictograms were presented twice within each subtask, which would allow for reliability analyses as well as for control purposes. Participants were randomly assigned to one of the four subtasks. As a result, 65, 60, 61, and 62 people participated, respectively, in each of the subtasks. After giving informed consent to participate in the study, participants provided demographic data, and read the instructions for the task on the computer screen. In each trial, a pictogram was presented in the center of the computer screen. Participants were instructed to type a single word that best described the meaning of that pictogram in a text box below it, allowing them to complete the task at their own pace. The word could be a verb, a noun, or an adjective. Prior to data analysis, misspelling errors in the participants’ responses were corrected by an independent native Spanish speaker.

The translucency task was also implemented using jsPsych (de Leeuw, 2015). The 1,525 stimuli were randomly divided into two subsets with 508 and one subset with 509 word-pictogram pairs each, in which the words were the ARASAAC labels for the pictograms. As in the transparency task, each subset was assigned to a separate translucency subtask and twenty word-pictogram pairs were presented twice, which would allow for reliability analyses as well as for control purposes. Additionally, in each subtask, twenty nonrelated word-pictogram pairs were presented along with the rest of the stimuli, randomly distributed, to detect potential inadequate performance in the translucency task. Participants were 87, 83, and 83, respectively, in each of the three subtasks. After giving informed consent to participate in the study, they provided demographic data, and read the instructions for their task on the computer screen. In each trial, participants had to rate the degree of relationship between the pictogram and the meaning of the word shown written below each pictogram, by clicking on a single number in a 7-point scale displayed below, where 1 represented no relationship between the pictogram and the meaning of the word, 4 meant that there was a moderate relationship, and 7 meant that the relationship was very strong. Verbal labels were shown at both extremes to remind participants of the scale values. The response time (i.e., the elapsed period between the onset of the word-pictogram pair on the screen and the click on the value scale) was registered for each trial.

## 3 Results and discussion

The supplementary material available at Open Science Framework^3^ includes a table that provides comprehensive details on the dataset, including the modal name (i.e., the most frequently provided name for each pictogram by the participants), the pictogram name according to ARASAAC, a comparison between the ARASAAC name and the modal name indicating whether or not there is an exact match between the two, a measure of the semantic relatedness between the ARASAAC name and the modal name, indicating how closely participants’ modal response align with the intended meaning, the image file names, the proportion of subjects that provided the ARASAAC name (i.e., transparency), two indices of naming consensus (name agreement scores and H scores), and the average of translucency rating scores. In the context of our study, name agreement refers to the proportion of participants who assigned the modal name to a specific pictogram (coinciding or not with the ARASAAC name), serving as an indicator of consistency in the identification and naming of pictograms among different individuals. On the other hand, the H statistic (Snodgrass and Vanderwart, 1980) measures the heterogeneity in naming responses, providing a numerical quantification of the variability in the names given to each pictogram. A low value in the H index indicates high name agreement, suggesting that most participants concurred on how to name a pictogram, while a high value signals greater diversity in assigned names, reflecting lesser consensus.

### 3.1 Descriptives

Table 1 presents the statistical summary for indicators of transparency (expressed as a proportion), name agreement and H, as well as for indicators of translucency (mean rating and mean response time) for the total set of pictograms, and for grammar-specific category subsets.

**Table 1:** Mean, standard deviation (SD), and the minimum and maximum values of transparency, name agreement, H statistic, translucency ratings, and translucency response times across all pictograms and separated by grammatical category of the ARASAAC name.

	Mean	SD	Min	Max
All pictograms (*N* = 1,525)				
Transparency	0.39	0.34	0	0.98
Name agreement	0.56	0.23	0.07	0.98
H	2.12	1.09	0.12	5.18
Translucency rating	6.33	0.82	1.75	6.99
Translucency response time (ms.)	2,815	650	1,845	8,223
Nouns (*n* = 1,194)				
Transparency	0.44	0.34	0	0.98
Name agreement	0.60	0.23	0.08	0.98
H	1.96	1.03	0.11	4.94
Translucency rating	6.46	0.71	1.76	6.99
Translucency response time (ms.)	2,713	601	1,845	6,380
Verbs (*n* = 301)				
Transparency	0.22	0.28	0	0.98
Name agreement	0.45	0.21	0.07	0.98
H	2.70	1.07	0.12	5.09
Translucency rating	5.87	1.03	1.75	6.95
Translucency response time (ms.)	3,184	699	2,076	8,222
Adjectives (*n* = 28)				
Transparency	0.21	0.23	0	0.68
Name agreement	0.44	0.21	0.10	0.86
H	2.78	1.10	0.96	5.18
Translucency rating	6.01	0.66	3.66	6.84
Translucency response time (ms.)	3,205	569	2,112	4,439
Adverbs (*n* = 2)				
Transparency	0.35	0.24	0.18	0.52
Name agreement	0.35	0.24	0.18	0.52
H	3.07	0.71	2.57	3.57
Translucency rating	6.50	0.24	6.33	6.67
Translucency response time (ms.)	2,672	763	2,132	3,212

**Figure 1:**
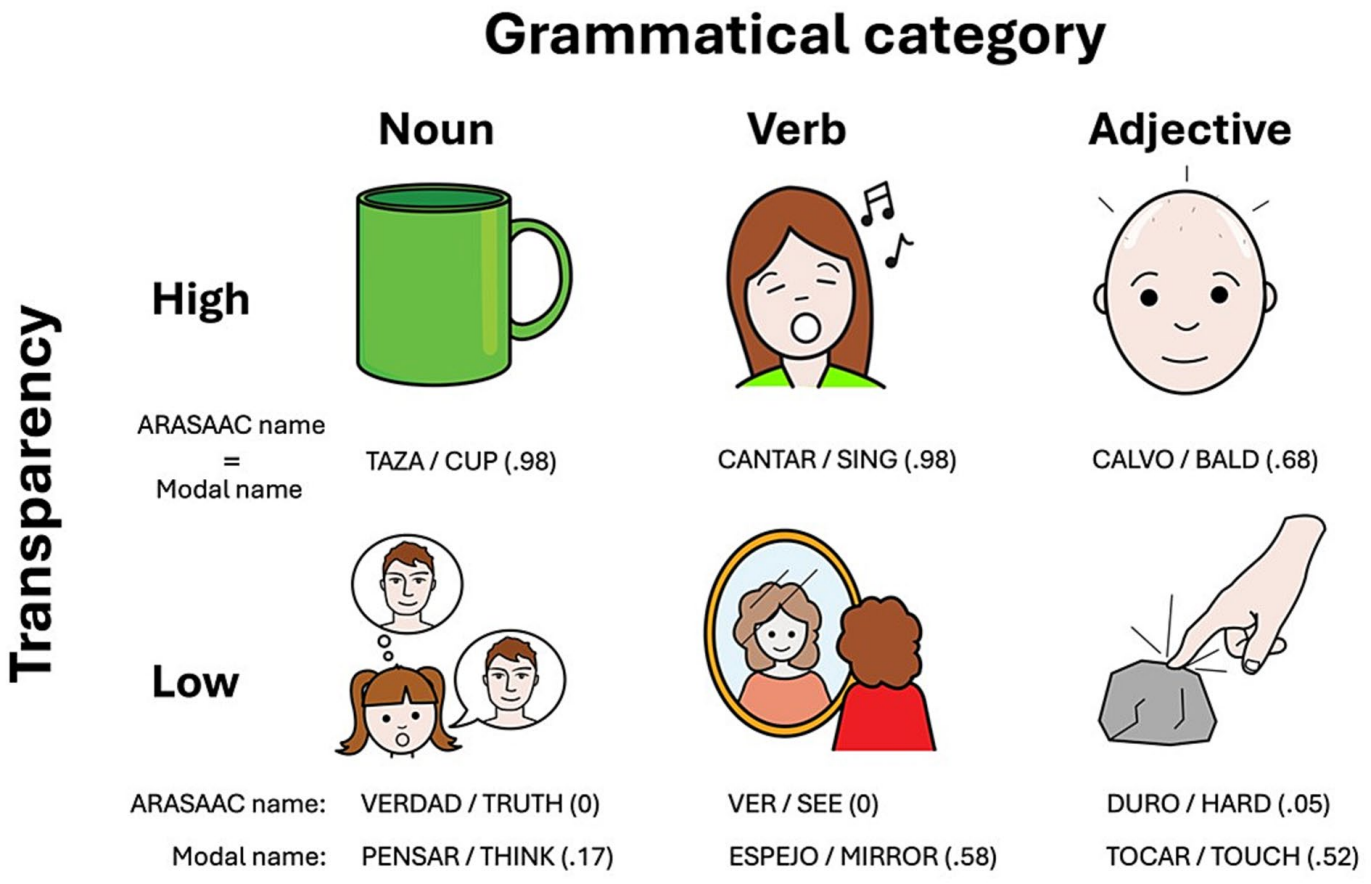
Examples of high and low transparency pictograms. In parentheses, the proportion of participants who provided the corresponding name. The pictographic symbols shown in this image are reproduced with permission from ARASAAC. The pictograms were created by Sergio Palao for ARASAAC, are property of the Aragón Government, and are distributed under a Creative Commons BY-NC-SA License.

The analysis of overall results in Table 1 revealed notable variations in the perception and cognitive processing of pictograms among participants. Specifically, both transparency and the degree of consensus on naming were relatively low, suggesting diverse interpretations and associations, which underscores the challenge in achieving uniformity in pictogram recognition. Figure 1 shows some examples of pictograms with high and low transparency from different grammatical categories. Conversely, the translucency ratings were consistently high, suggesting a widespread agreement that pictograms presented with a plausible name label are significantly related.

Exploring further the overall results according to grammatical category, nouns, which constitute the majority with 1,194 instances, show higher transparency (*M* = 0.44), name agreement (*M* = 0.60), and slightly more consistency (lower H index of 1.96) than adjectives [*t*(1,521) = −3.67, *p* < 0.01; *t*(1,521) = −3.64, *p* < 0.01; *t*(1,521) = 4.09, *p* < 0.001] and verbs [*t*(1,521) = 10.47, *p* < 0.001; *t*(1,521) = 9.78, *p* < 0.001; *t*(1,521) = −10.98, *p* < 0.001]. This reflects a stronger consensus in naming nouns and less variability among respondents. Nouns also score high on translucency ratings (*M* = 6.46), significantly higher than verbs [*t*(1,521) = 11.70, *p* < 0.001] and adjectives [*t*(1,521) = −2.95, *p* < 0.05], and also exhibit shorter average response times (*M* = 2,713 ms.) than verbs [*t*(1,521) = −11.8, *p* < 0.001] and adjectives [*t*(1,513) = 4.15, *p* < 0.001], indicating a possibly more straightforward cognitive processing. On the other hand, verbs demonstrate lower transparency (*M* = 0.22), lower name agreement (*M* = 0.45) and higher heterogeneity (H index = 2.70) than nouns, underscoring the complexity and variability in conceptualizing and naming actions. The translucency ratings for verbs average 5.87, significantly lower than the average for noun rating, and their evaluation required relatively long response times (*M* = 3,184 ms.), what may reflect the additional cognitive effort needed to process these word-pictogram pairs. Finally, adjectives show low transparency (*M* = 0.21) and name agreement (*M* = 0.44), and a high degree of heterogeneity (H index = 2.78), indicating considerable variability in how qualities are visually represented and recognized. Their translucency ratings (*M* = 6.01) are relatively high. The results of the adverbs are not discussed because there were only two exemplars in the database.

Further analysis involved comparing the modal names with the names provided by ARASAAC for each pictogram. Among the 1,525 pictograms, we observed discrepancies in 713 cases (53.2%), indicating a substantial difference between pre-assigned names and empirically-derived names. Table 2 details the variations in transparency, name agreement, H statistic, translucency ratings, and translucency response times between the two pictogram groups.

**Table 2:** Statistical summary of transparency, name agreement, H statistic, translucency ratings, and translucency response times as a function of name coincidence with ARASAAC.

	Name coincidence with ARASAAC			
	Yes (*n* = 812)		No (*n* = 713)	
	Mean	SD	Mean	SD
Transparency	0.66	0.21	0.08	0.10
Name agreement	0.66	0.20	0.45	0.20
H	1.67	0.94	2.64	1.01
Translucency rating	6.73	0.25	5.87	0.98
Translucency response time (ms.)	2,494	394	3,180	691

Pictograms that received names that coincide with the ARASAAC names demonstrated higher mean transparency [Welch’s *t*(1176.4) = −69.76, *p* < 0.001, *d* = −3.5] and higher name agreement [Welch’s *t*(1512.6) = −20.07, *p* < 0.001, *d* = −1.03], alongside a lower H statistic [Welch’s *t*(1462.6) = 19.36, *p* < 0.001, *d* = 1.0], suggesting greater consistency in naming within this subset. Additionally, these pictograms were rated as more translucent, with a mean translucency rating of 6.73 [Welch’s *t*(794.9) = −22.69, *p* < 0.001, *d* = −1.20], and the associated response times were faster, averaging at 2,494 ms. [Welch’s *t*(1097.7) = 23.37, *p* < 0.001, *d* = 1.22].

Given the discrepancies observed between the names provided by ARASAAC and the modal names produced by participants, it was interesting to determine whether these differences reflected underlying semantic relationships. To address this, we conducted an analysis of semantic similarity between the ARASAAC names and the obtained modal names. This analysis allowed us to explore if, despite the lack of exact matches, there were significant semantic connections between the terms.

We used a pre-trained Word2Vec model (Almeida and Bilbao, 2018) to represent words as vectors in a high-dimensional space. Then, we computed the cosine similarity between the vectors corresponding to each ARASAAC-modal name pair, which served as a measure of their semantic proximity (from −1 to +1, where +1 indicates maximum similarity, 0 indicates no similarity, and − 1 represents complete opposition).

The results revealed distinct patterns. For nouns, the average semantic similarity was 0.43, with a minimum of −0.08 and a maximum of 0.94, indicating a relatively high degree of semantic alignment. In the case of verbs, the average similarity was lower, at 0.31, with a minimum of −0.11 and a maximum of 0.86, suggesting greater variability in action-related terms. Finally, for adjectives, the average similarity was 0.30, ranging from 0.08 to 0.63, reflecting moderate semantic connections. These findings demonstrate that nouns tend to have higher semantic similarity compared to verbs and adjectives, which may relate to their more concrete and visually representable nature, facilitating better alignment with the pictograms.

### 3.2 Reliability

The reliability of the transparency ratings was evaluated in various ways. As explained above, 20 pictograms were presented twice within each of the four subtasks (making a total of 80 repeated stimuli), allowing for reliability analyses. Initially, correlations between the name agreement indices for the group of 80 repeated pictograms were calculated. There was a total match in the most frequent response for the majority of the 80 repeated pictograms (94%). Both the correlation of name agreement (*r* = 0.98, *p* < 0.001) and the H statistic (*r* = 0.99, *p* < 0.001) reached very high and significant values. Additionally, the ratings were divided into two random halves, and the ICC between the two halves was calculated. Both in the case of name agreement [ICC(2,k) = 0.99; 95% CI: 0.99–1.00] and the H index [ICC(2,k) = 0.99; 95% CI: 0.99–1.00], the results showed high consistency. Overall, these results show high consistency in the responses of the transparency task.

The reliability of judgments in the translucency task was assessed in three ways: through the correlation of scores assigned to word-pictogram pairs that were presented repeatedly within the task, evaluating judgments for mismatched word-pictogram pairs, and by calculating the intraclass correlation coefficient (ICC2k, average random raters) between two random halves of all judgments. First, results demonstrated a significant and positive correlation between scores for repeated word-pictogram pairs (*r* = 0.99; *p* < 0.001), indicating a high consistency in participants’ translucency judgments. Second, the mean translucency rating for the 60 mismatched word-pictogram pairs reached a low value of 1.49 (*SD* = 0.63), as expected for random word-pictogram pairs, denoting the participants’ adherence to the rating task. Last, the ICC between the two halves revealed excellent reliability [ICC(2,k) = 0.98; 95% CI: 0.97–0.98], suggesting the stability and accuracy of the judgments made in the translucency task. Overall, these findings suggest that the translucency judgments provided by participants can be considered reliable.

### 3.3 Validity

Validity was assessed by calculating several consistency measures between responses to a subset of 191 pictograms shared with the transparency norms provided by Paolieri and Marful (2018).

First, the correlation between name agreement and H for the set of shared stimuli that led to the same modal response (*n* = 126) was calculated. Both the correlation in name agreement (*r* = 0.73, *p* < 0.001) and in the H statistic (*r* = 0.76, *p* < 0.001) reached significant and moderately high values.

Second, we calculated the Hellinger affinity (HA) between our norms and oral and written norms obtained by Paolieri and Marful (2018). The HA quantifies the extent of overlap between two distributions, with values spanning from 0 (denoting no overlap) to 1 (indicating complete congruence between the distributions). To achieve this, HA scores were calculated (for details on the calculation, see Paolieri and Marful, 2018) for all pictograms shared across both studies. The results revealed that the overlap with written norms (*HA* = 0.64, *SD* = 0.27) and oral norms (*HA* = 0.68, *SD* = 0.27) reached moderately high values, and a significant number of words exhibited values greater than 0.5 in both modalities (78% and 80%, respectively).

Overall, the validity results demonstrate robust consistency in the perception and naming of pictograms among participants from both studies, as well as substantial congruence between the naming distributions of the two normative studies, thereby supporting the validity of the norms obtained in the current study.

### 3.4 Exploring the relationships between transparency and translucency and other variables of interest

By analyzing the intricate interplay between a pictogram’s visual properties (such as symmetry or complexity) and the multifaceted representational dimensions of its referents (lexical, semantic, sensory, motor, and emotional), along with transparency and translucency measures, fundamental elements that may contribute to its communicative effectiveness can be uncovered. To achieve this, we collected a set of subjective and objective indicators related to both visual aspects of the pictogram and the most frequent verbal labels used to describe their referents.

For the pictogram images, we computed various indices to assess their processing fluency and visual complexity. We used the *imagefluency* library (Mayer, 2024), which applies processing fluency theory to calculate scores for key aesthetic principles that influence how easily images are processed cognitively. These principles include contrast, complexity/simplicity, self-similarity, symmetry, and typicality. As detailed by Mayer and Landwehr (2018), these features contribute to the ease of image processing, ultimately impacting perception and interpretation. Additionally, we quantified pictogram complexity by measuring image entropy (amount of information or variability in the visual patterns) and edge density (total edges divided by image area). These measures provide insights into the visual structure of the pictograms, aiding in understanding their interpretability and cognitive demands.

For the set of ARASAAC names of the pictograms, data from several variables were obtained for the shared stimuli with other databases, related to multiple dimensions, namely lexical: written frequency (Duchon et al., 2013) and age of acquisition (Alonso et al., 2016; Alonso et al., 2015); semantic: familiarity, imageability, concreteness (Duchon et al., 2013), and semantic density (Díez et al., 2018; Fernandez et al., 2004); emotional: valence and arousal (Stadthagen-Gonzalez et al., 2017); motor: body object interaction (BOI) (Alonso et al., 2018); sensory: sensory experience ratings (SER; Díez-Álamo et al., 2019); seven more specific perceptual and motor attributes (Díez-Álamo et al., 2018) and lexical decision times (González-Nosti et al., 2014).

**Figure 2:**
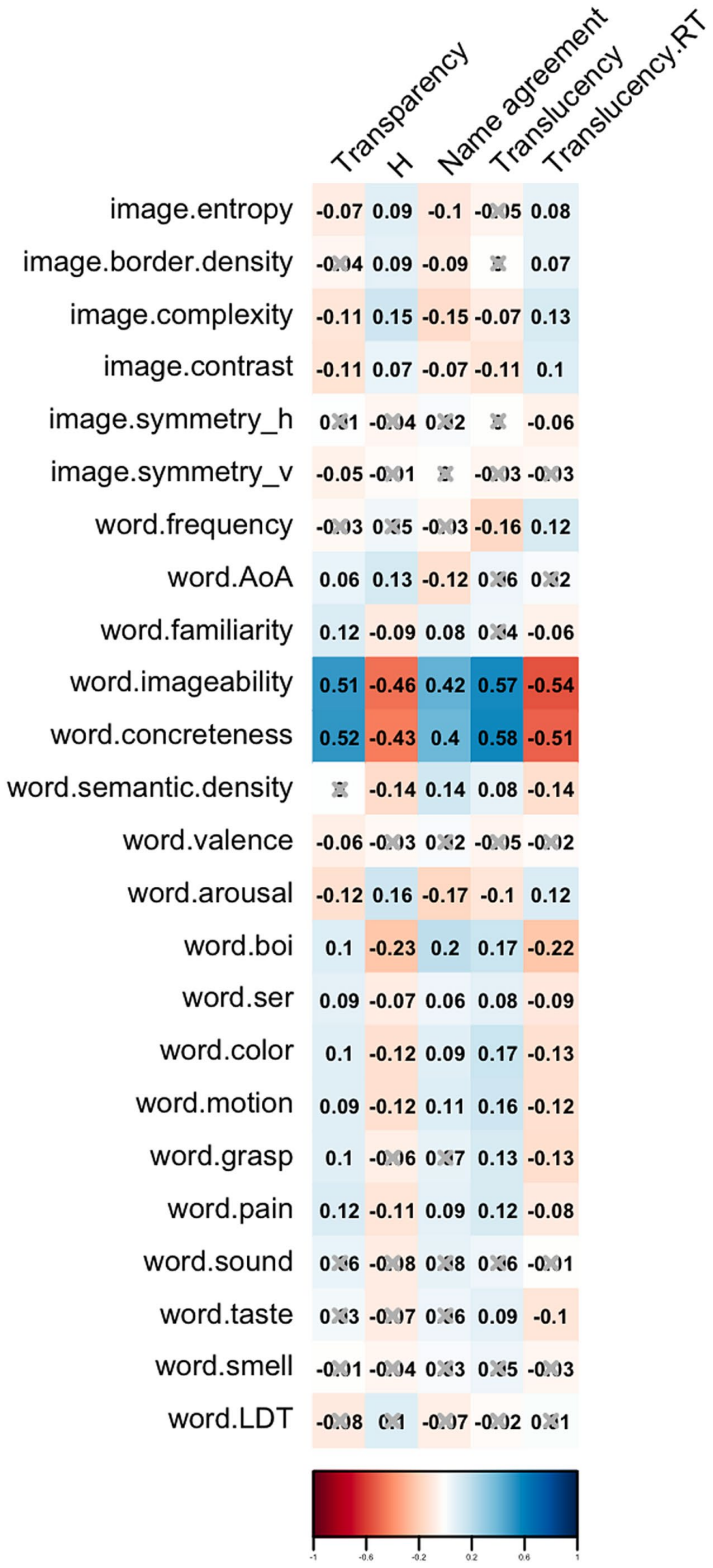
Correlogram showing Spearman correlations between image properties, ARASAAC name properties, and indicators of transparency, name agreement, H index, and translucency (mean rating and mean response time).

As shown in Figure 2, imageability and concreteness correlated positively with both transparency and translucency. This suggests that pictograms associated with concepts that are more tangible and easily visualized are perceived as markedly more transparent and translucent.

Visual characteristics of pictograms such as entropy, border density, image complexity, and image contrast exhibited low degrees of correlation with transparency, translucency and naming consensus, indicating the modest impact that these visual factors appear to have on the interpretation and agreement of pictogram representations.

Lastly, the emotional connotations of words, captured by valence and arousal indices, showed weak or negligible correlations with transparency, name agreement and translucency.

### 3.5 Differential attribute importance for name match with ARASAAC

**Figure 3:**
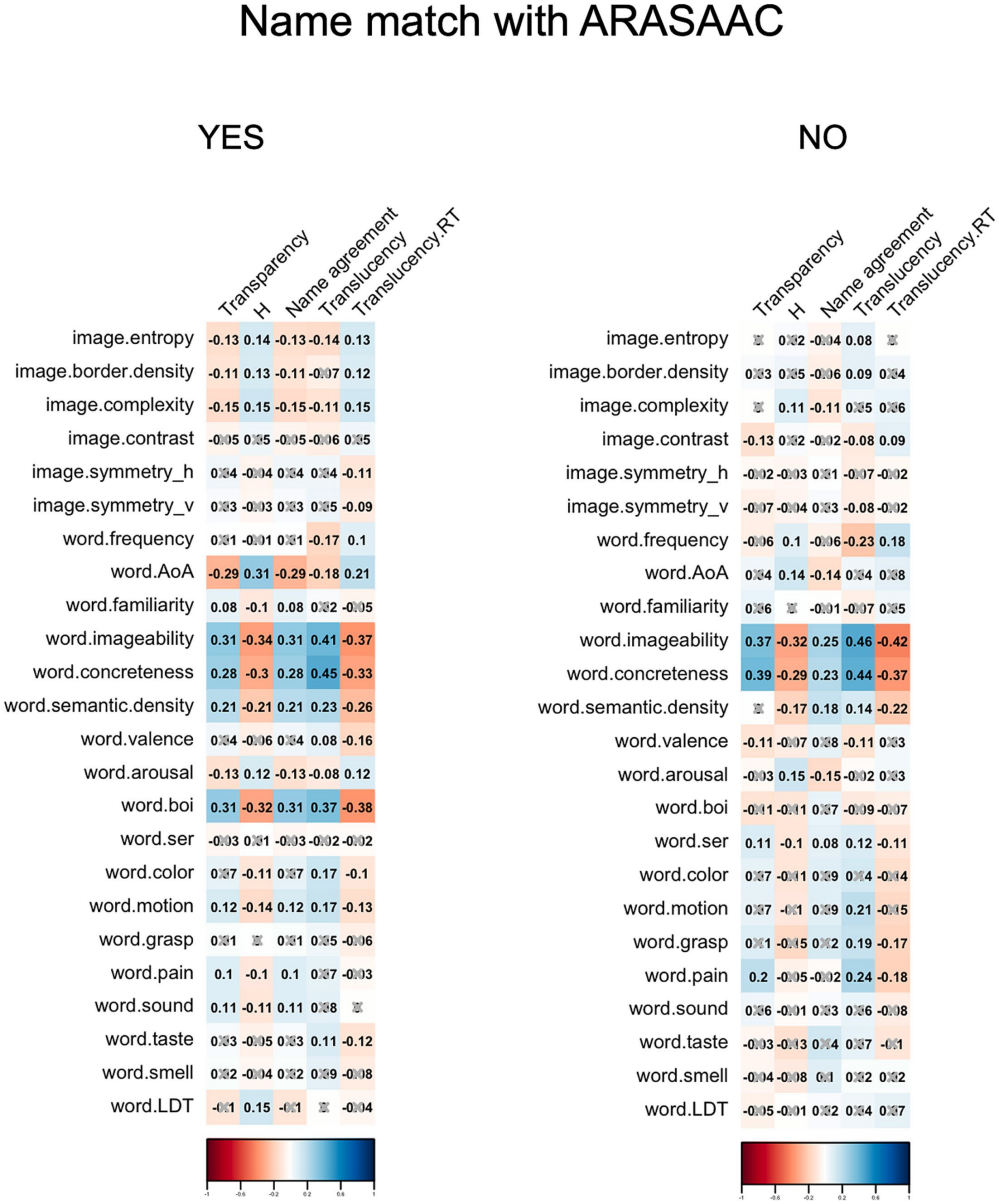
Correlograms showing Spearman correlations between image and modal name properties and indicators of transparency and translucency as a function of modal name match with ARASAAC.

Given the widespread application of ARASAAC pictograms in educational and therapeutic settings for individuals with communication challenges, another interesting issue to explore is the identification of the differential factors related to increases in transparency and translucency values based on whether the most frequently provided name (i.e., the modal name) matches the one originally assigned by the designers. To this end, Figure 3 displays the Spearman correlations between the set of image and word features and the transparency, name agreement and translucency indexes, depending on whether there is a match or not. Only exact coincidences were considered as matches.

As seen in Figure 3, some image or referent variables exhibited significant correlations only when there is a name match with ARASAAC. Concerning variables linked to pictograms with matching modal names, factors such as age of acquisition (with later acquired words linked to lower transparency and translucency) or the degree of body-object interaction (with higher values associated with greater transparency and translucency) emerged as relevant for matching pictograms. Similarly, various image variables such as entropy, complexity, and border density correlated negatively with transparency and translucency values, albeit the correlation values are low.

To further explore the attributes of pictograms that are associated with name match, an additional analysis was conducted comparing the results of two different variable selection techniques to build a prediction model: Boruta (Kursa and Rudnicki, 2010) and least absolute shrinkage and selection operator (LASSO) (Tibshirani, 1996). The Boruta feature selection algorithm is a non-parametric, random forest-based approach, adept at managing intricate interactions and nonlinear relationships among features, proving to be an especially advantageous way of navigating through high-dimensional datasets or elaborate arrays of potential predictors. LASSO is a regularization technique used in linear regression models to enhance prediction accuracy and model interpretability. By adding an L1 penalty term to the loss function, LASSO shrinks the coefficients of less important variables to exactly zero, effectively performing variable selection. This results in simpler models that avoid overfitting and highlight the most significant predictors, making LASSO particularly useful in high-dimensional datasets where the number of predictors is large.

A systematic approach to both train and test the model was used to ensure robustness and reliability in its predictive capabilities. Initially, the dataset (1,525 rows) was partitioned into two distinct subsets: a training set, comprising 70% of the data, designated for model training, and a testing set, constituting the remaining 30%, used for model evaluation. This division was facilitated through random sampling ensuring that both sets contained representative examples of match/no match pictograms. Then, we used the Boruta algorithm and LASSO regression with cross-validation on the training set to identify important variables with both methods. Last, we trained two classification models (logistic regression) using the sets of variables selected by Boruta and LASSO and evaluated the two models on the test set using accuracy, precision, sensitivity, specificity, and the area under the ROC curve (ROC AUC).

Based on the evaluation metrics, both models showed an acceptable discriminative capacity between matching and non-matching pictograms, but the model using variables selected by Boruta demonstrated slightly better performance. The Boruta-based model achieved an accuracy of 0.75, a precision of 0.76, a sensitivity of 0.66, a specificity of 0.82, and an ROC AUC of 0.79. In comparison, the LASSO-based model achieved an accuracy of 0.72, a precision of 0.73, a sensitivity of 0.64, a specificity of 0.79, and an ROC AUC of 0.78. The higher accuracy, precision, specificity, and ROC AUC of the Boruta-based model indicate its superior discriminative ability, and consequently, the Boruta-selected variables were considered for discussion.

**Figure 4:**
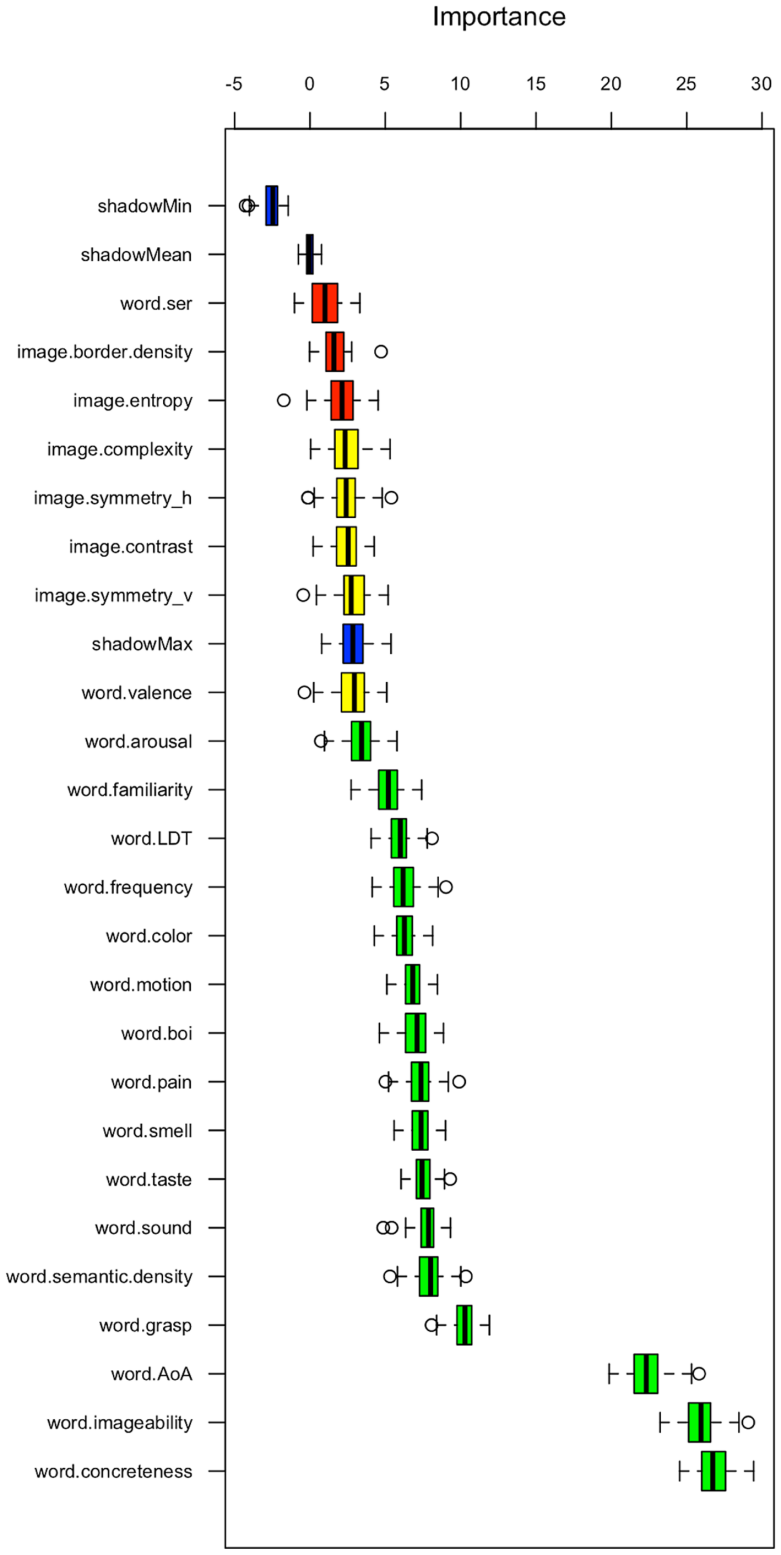
Boruta derived feature importance for name match with ARASAAC.

Figure 4 displays the importance of each feature for name match with the ARASAAC original labels of the pictograms. The results suggested that all features were deemed important; however, word imageability, word concreteness, and word age of acquisition (AoA) demonstrated notably higher importance scores than the others. Therefore, pictograms with names characterized by high imageability and concreteness, along with those learned early in life, are more likely to achieve name matching with ARASAAC, owing to their clear, tangible depictions and broad recognition.

## 4 Final conclusions

This study has provided an extensive analysis of transparency and translucency indices across a large set of 1,525 pictograms from the ARASAAC database, with an emphasis on the interaction between visual properties of the pictograms and the linguistic characteristics of their word referents. This research stands as the most comprehensive study conducted on the transparency and translucency of ARASAAC pictograms to date. By examining a set of visual and linguistic attributes across a significant number of pictograms, we also have delved deeply into how these factors interplay to influence pictogram perception and understanding.

From a methodological standpoint, the study’s reliability was bolstered by consistent name agreement and translucency ratings across repeated measures and participants. The robustness of these findings is further supported by the alignment with the findings of previous research (Paolieri and Marful, 2018), thereby confirming the validity of the measures used in this study.

Overall, our findings suggest that pictograms that are named consistently with ARASAAC original labels are perceived more uniformly and processed more efficiently, highlighting the potential cognitive advantages of standardization in pictogram nomenclature. The substantial differences in transparency, naming agreement, H statistic, and translucency perceptions underscore the importance of considering empirically derived norms when selecting pictograms for communication purposes. It is noteworthy that the average transparency of the studied pictograms is below the ISO standard criteria, even for the group of pictograms whose modal names matched those of ARASAAC. It should be kept in mind that only exact matches with ARASAAC names were taken into account for the calculation of transparency indices. However, our analysis revealed that even when the terms provided by participants did not exactly match the ARASAAC labels, significant semantic relationships often existed. Future research should further explore these connections, particularly examining the impact of semantic relationships such as hyponymy and hypernymy, as well as ambiguity, on pictogram interpretation to enhance the cognitive accessibility and effectiveness of pictograms.

This study also shed light on the differential processing of pictograms according to grammatical category. Nouns exhibited higher transparency, name agreement and consistency, suggesting that objects and other noun referents are named and recognized with greater uniformity than actions or qualities, as represented by verbs and adjectives, respectively. These results are consistent with previous research (Bloomberg et al., 1990; Haupt and Alant, 2002; Lloyd and Fuller, 1990; Mizuko, 1987; Mizuko and Reichle, 1989), which has shown that nouns, being more concrete and visually representable, tend to be more transparent and cognitively accessible than verbs. These variations underscore the importance of considering grammatical categories when considering the adequacy of pictograms for communication purposes. Also, in line with recent work (Horno-Chéliz, 2024; Ivanova, 2024), which reviews important studies on discreteness versus graduality in the processing of grammatical categories, our findings, from a different perspective but with converging results, suggest that nouns are more cognitively stable, while verbs and adjectives present greater challenges in processing and accessibility.

Our results interestingly show that, for both transparency and translucency, word imageability and concreteness were the most correlated factors. These dimensions, along with the age of acquisition, were also identified as the most important features related to the matching of modal names with ARASAAC labels. This indicates that pictograms with names characterized by high imageability and concreteness, and those learned at an earlier age, are more likely to receive names that align with the names assigned by their creators. This relationship underscores the significance of these linguistic features in the perception and naming of pictograms, highlighting their role in achieving name matching with ARASAAC. Also, this result aligns with the intuitive understanding that a pictogram’s effectiveness is significantly determined by the ease with which its associated word can evoke a mental image or sensory experience.

In summary, this study has the potential to enrich both the theoretical understanding of pictogram processing and more practical approaches to pictogram design by highlighting attributes critical to pictogram comprehension. It not only provides guidelines for the creation of more effective and universally understood pictograms, but it also introduces a comprehensive pictogram database for enhancing design and communication efficacy. This database, detailing the most frequent denominations as well as transparency and translucency indices, constitutes a powerful tool to support informed pictogram selection and application, thereby becoming critical towards improving visual communication and cognitive accessibility. Ultimately, these contributions foster pictogram comprehension and facilitate data-driven decision-making in pictographic system development, potentially enhancing cognitive accessibility for individuals with communication challenges.

## Data Availability

The dataset presented in this study can be found at Open Science Framework: https://osf.io/eyjr6/.

## Footnotes

^1^ http://arasaac.org

^2^Please note that the ARASAAC catalog is subject to updates.

^3^ https://osf.io/eyjr6/
